# Transient Postoperative Diabetes Insipidus With Hypovolemic Shock Temporally Associated With Dexmedetomidine, Propofol, and Sevoflurane: A Case Report

**DOI:** 10.1002/ccr3.72738

**Published:** 2026-05-17

**Authors:** Yuan Meng, Xiaoxiao Qu, Yue Jin, Chengwei Yin, Geping Zhang, Fang Xie, Rong Shi

**Affiliations:** ^1^ Department of Critical Care Medicine, Shuguang Hospital Shanghai University of Traditional Chinese Medicine Shanghai China

**Keywords:** case report, dexmedetomidine, diabetes insipidus, hypovolemic shock, propofol, sevoflurane

## Abstract

Dexmedetomidine, propofol, and sevoflurane are widely used anesthetic agents, but reports of water metabolism disturbances—particularly drug‐induced diabetes insipidus (DI)—associated with their use remain extremely rare. We report a case of a 51‐year‐old Chinese man who developed abrupt high‐volume polyuria (> 600 mL/h; 24‐h output of 8750 mL), hypotonic urine (specific gravity 1.003; osmolality 175 mOsm/kg), progressive hypernatremia (peak 156 mmol/L), hemoconcentration, lactic acidosis, and hypotension following transurethral seminal vesiculoscopy performed under general anesthesia. The patient was initially suspected of having an anaphylactic reaction, but standard interventions were ineffective. After exclusion of other causes, a diagnosis of anesthetic‐associated DI with secondary hypovolemic shock was made. The patient responded to aggressive fluid and electrolyte replacement alongside norepinephrine support. Polyuria resolved within approximately 18 h, serum sodium normalized, and the patient was discharged from the ICU on postoperative Day 3. This case emphasizes that persistent dilute polyuria with rising serum sodium after anesthesia should prompt early evaluation for DI. When such complications arise, timely diagnosis, targeted fluid management, and hormone replacement therapy when appropriate can be crucial for preventing potentially life‐threatening outcomes.

## Key Clinical Message

Transient diabetes insipidus may rarely occur after exposure to anesthetic agents, particularly in the setting of combined drug exposure. In patients with unexplained postoperative polyuria, hypernatremia, and circulatory instability, early recognition of hypotonic water diuresis is essential to prevent severe hypovolemia and shock.

## 1 Introduction

Diabetes insipidus (DI) is a disorder characterized by impaired urinary concentrating ability due to insufficient secretion of antidiuretic hormone (ADH, also known as arginine vasopressin, AVP) or renal resistance to ADH. Clinically, DI presents with polyuria and polydipsia, with daily urine volumes often exceeding 50 mL/kg [1]. In the perioperative setting, DI is extremely uncommon [2]. Most reported cases are associated with neurosurgery, pituitary disease, or severe head trauma. Reports implicating commonly used anesthetic agents in the development of DI are rare but have been published. When unrecognized, drug‐induced DI may progress to hypernatremic hypovolemia and be mistaken for distributive shock, delaying appropriate fluid and vasopressin therapy. We describe a case of transient postoperative DI with hypovolemic shock that developed shortly after combined exposure to dexmedetomidine, propofol, and sevoflurane.

## 2 Case History/Examination

A 51‐year‐old man weighing 70 kg was admitted with a 3‐month history of hemospermia. His past medical history was notable for type 2 diabetes mellitus controlled with metformin. He had no history of head trauma, intracranial pathology, pituitary disease, prior surgeries, or drug allergies. Preoperative laboratory tests, including complete blood count, renal and liver function, coagulation studies, electrolytes, and ECG, were within normal limits. Preoperative serum sodium: 141 mmol/L; glucose: 6.6 mmol/L.

### 2.1 Perioperative Course

The patient underwent transurethral seminal vesiculoscopy with lithotripsy under general anesthesia. Total anesthesia time was 50 min and operative time was 40 min. Intraoperative medications included intranasal dexmedetomidine 30 μg, dezocine 10 mg, propofol 130 mg (1.86 mg/kg), dexamethasone 5 mg, mivacurium 13 mg, sevoflurane, and palonosetron hydrochloride 0.25 mg. Intraoperative fluid administration consisted of 500 mL lactated Ringer's solution. Hemodynamics remained stable during the procedure, with blood pressure around 95–100/60–65 mmHg.

Approximately 0.9 h after completion of anesthesia, while in the post‐anesthesia care unit (PACU), arterial blood gas analysis was obtained because the patient remained somnolent and hypotensive after an otherwise uneventful short procedure. At that time, blood pressure was 80/50 mmHg. Physical examination showed somnolence but arousable mental status; no rash, wheezing, or airway edema. Arterial blood gas showed pH 7.284, HCO_3_^−^ 18.7 mmol/L, and lactate 6.4 mmol/L. Hypovolemia or early shock was suspected. Over the next 2 h, 700 mL lactated Ringer's solution and 200 mL 5% sodium bicarbonate were administered, during which urine output was already 600 mL.

At approximately 2.3 h after anesthesia, the patient was returned to the ward, still somnolent but arousable. By approximately 5.9 h, he became agitated and developed mottling of the lower limbs and abdomen. Repeat blood gas analysis showed pH 7.052, HCO_3_^−^ 10.2 mmol/L, lactate 22 mmol/L, and Na^+^ 144 mmol/L, prompting transfer to the ICU.

At approximately 7.0 h after anesthesia, arterial blood gas in the ICU showed pH 7.098, HCO_3_^−^ 12.6 mmol/L, Na^+^ 152 mmol/L, and lactate 27 mmol/L. Bedside ultrasound demonstrated collapsed jugular veins, and central venous pressure was approximately 5 cmH_2_O. Review of the recorded urine output showed persistent polyuria exceeding 600 mL/h, totaling approximately 3000 mL over 4.5 h despite no diuretic exposure. Rapid crystalloid infusion (800 mL/h) and norepinephrine at 0.5 μg/kg/min were initiated. Sodium bicarbonate was used as a temporizing measure in the setting of severe acidemia while the primary treatment focus remained restoration of intravascular volume and tissue perfusion.

By approximately 14.8 h after anesthesia, the patient had regained full consciousness, reported intense thirst, and was able to drink orally. Fluid administration was gradually reduced and norepinephrine was tapered. During the first 24 postoperative hours, total intake was 7447 mL (5747 mL crystalloids, 500 mL colloids, and 1200 mL sodium bicarbonate) plus 1200 mL oral fluids, while urine output reached 8750 mL. Laboratory tests showed hemoconcentration (hemoglobin 187 g/L, preoperative 145 g/L), hyposthenuria (urine specific gravity 1.003, urine osmolality 175 mOsm/kg), and renal impairment (serum creatinine 130 μmol/L). By the following afternoon, hemodynamics had improved and norepinephrine was discontinued.

## 3 Differential Diagnosis, Investigations, and Treatment

On ICU admission, the dominant problem was circulatory shock. The differential diagnosis included hypovolemic shock, anaphylaxis, cardiogenic shock, obstructive shock, postoperative hemorrhagic shock, procedure‐related complications such as perforation, and postoperative sepsis/septic shock. Physical examination at that stage showed mottling of the lower limbs and abdomen, low filling status on bedside assessment, and no evidence of hypoxemia. Bedside echocardiography showed preserved cardiac contractility, no pericardial tamponade, and a relatively small right ventricle, making cardiogenic and obstructive causes less likely. Low central venous pressure and collapsed jugular veins favored intravascular volume depletion.

Postoperative hemorrhage was specifically considered because of the abrupt hemodynamic deterioration after an endoscopic urologic procedure. However, this was considered less likely because there was no documented massive hematuria or other overt bleeding source, and hemoglobin increased rather than decreased during the shock phase, consistent with hemoconcentration rather than blood loss. Irrigation‐related complications were also considered, but the biochemical pattern was not typical, as serum sodium rose progressively instead of falling. Procedure‐related perforation or other surgical complications were also part of the differential diagnosis. The patient had no abdominal distension, abdominal tenderness, peritoneal signs, pelvic pain, or other localizing findings suggestive of perforation, and Abdominal CT scan on the following day was normal.

Osmotic diuresis due to hyperglycemia was considered but was less likely. During the period of maximal polyuria, blood glucose ranged from 9.5 to 15.6 mmol/L, urine ketones were negative, and the urine remained markedly dilute, with a specific gravity of 1.003 and osmolality of 175 mOsm/kg. This pattern is more consistent with hypotonic water diuresis than solute diuresis, which typically presents with a higher urine osmolality.

**FIGURE 1:**
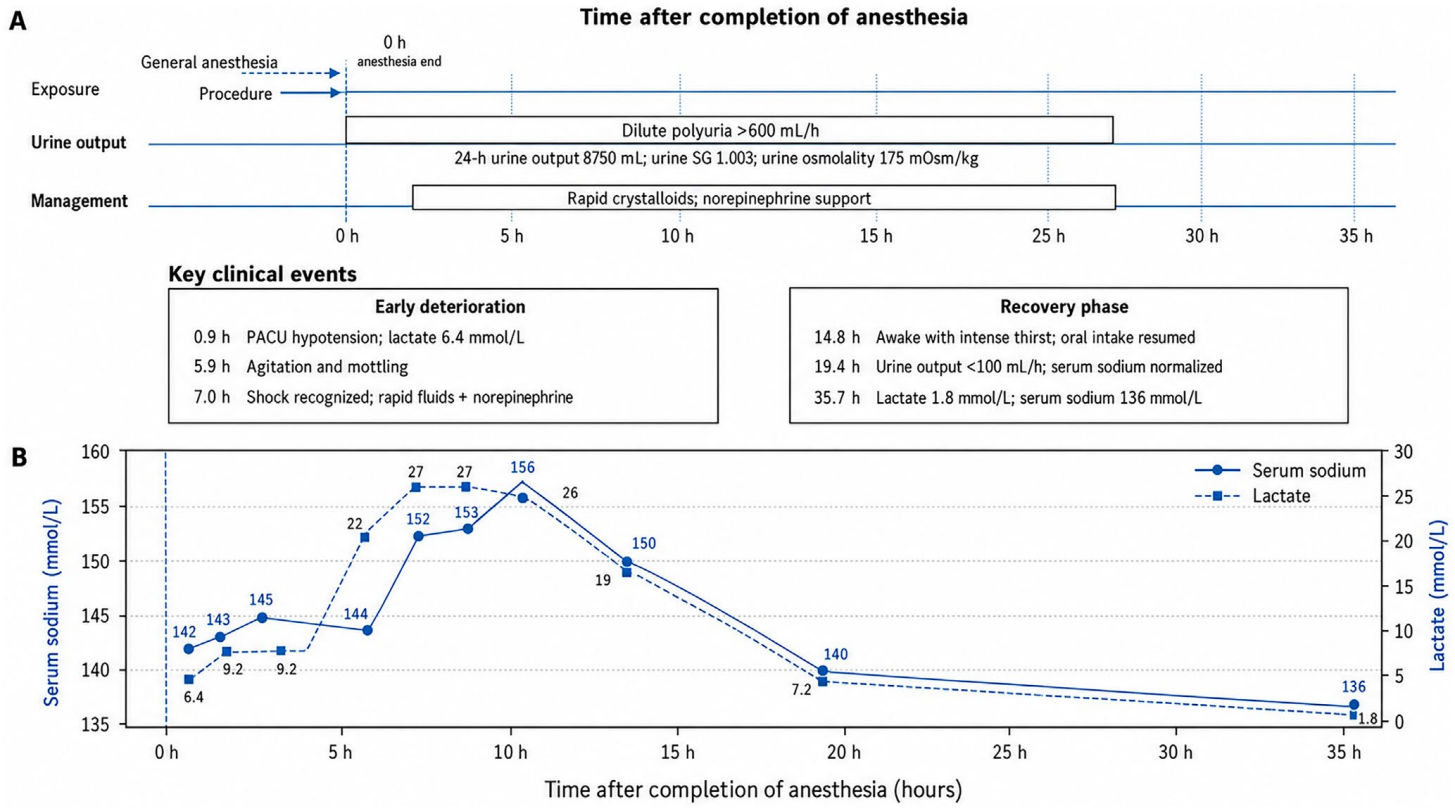
The timeline is expressed as hours after completion of anesthesia, which was defined as 0 h. (A) Summarizes perioperative exposure, the onset and duration of dilute polyuria, and major therapeutic interventions. General anesthesia and the procedure occurred before 0 h. Persistent dilute polyuria developed shortly after anesthesia, with urine output exceeding 600 mL/h and a 24‐h urine output of 8750 mL. Urine specific gravity was 1.003 and urine osmolality was 175 mOsm/kg. Rapid crystalloid infusion and norepinephrine support were initiated after shock was recognized. (B) Shows serial changes in serum sodium and lactate. Serum sodium increased to a peak of 156 mmol/L, while lactate increased to 27 mmol/L during the shock phase, followed by gradual normalization after fluid resuscitation and supportive treatment. PACU, post‐anesthesia care unit; SG, specific gravity.

**FIGURE 2:**
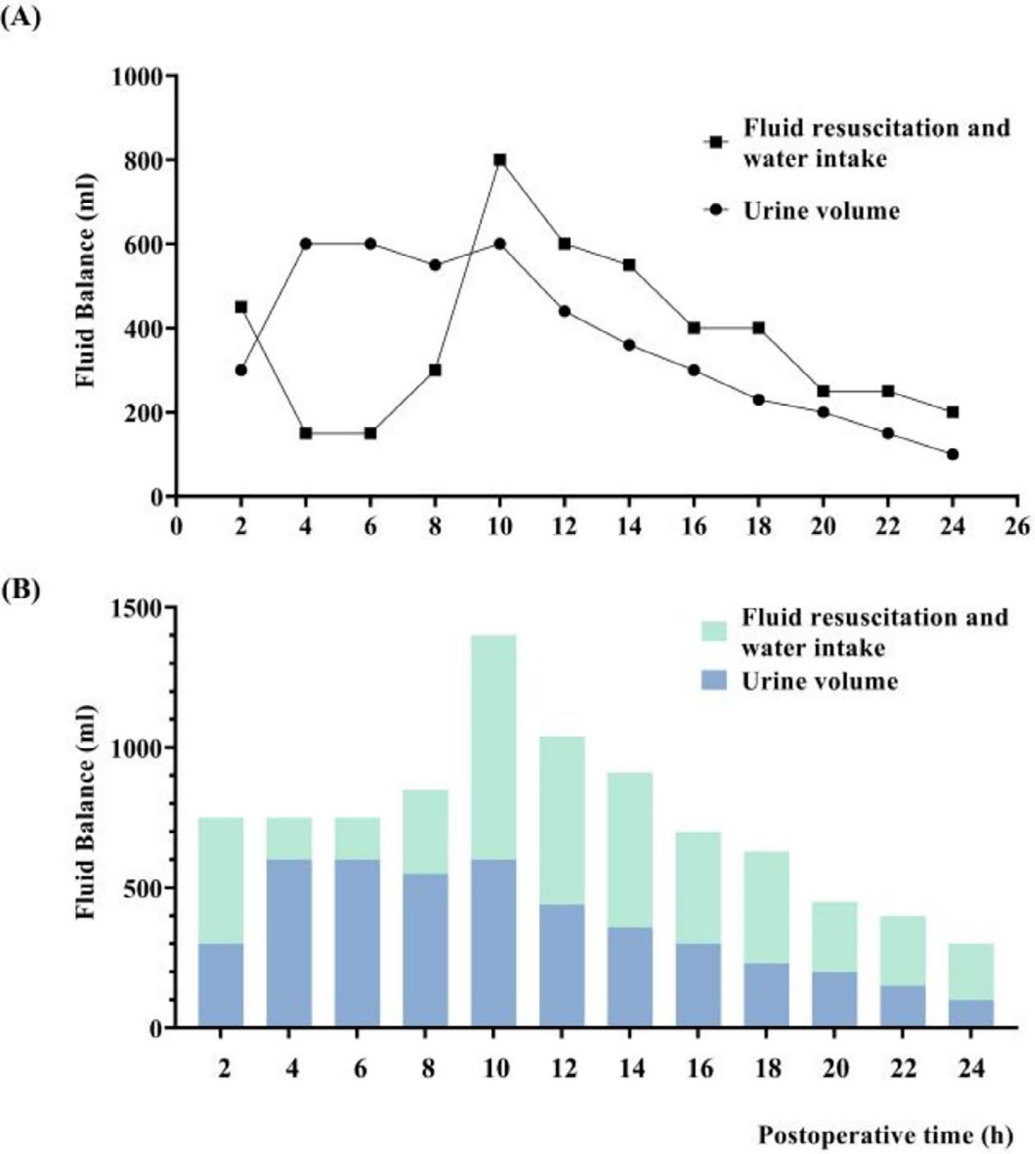
Postoperative fluid volume and urine output during the first 24 h after surgery. (A) Line graph showing changes in fluid resuscitation plus oral water intake and urine output at 2‐h intervals. (B) Stacked bar chart showing the temporal distribution of fluid resuscitation plus oral water intake and urine output. Marked postoperative polyuria was observed in the early postoperative period and gradually improved after aggressive fluid replacement and hemodynamic support.

**FIGURE 3:**
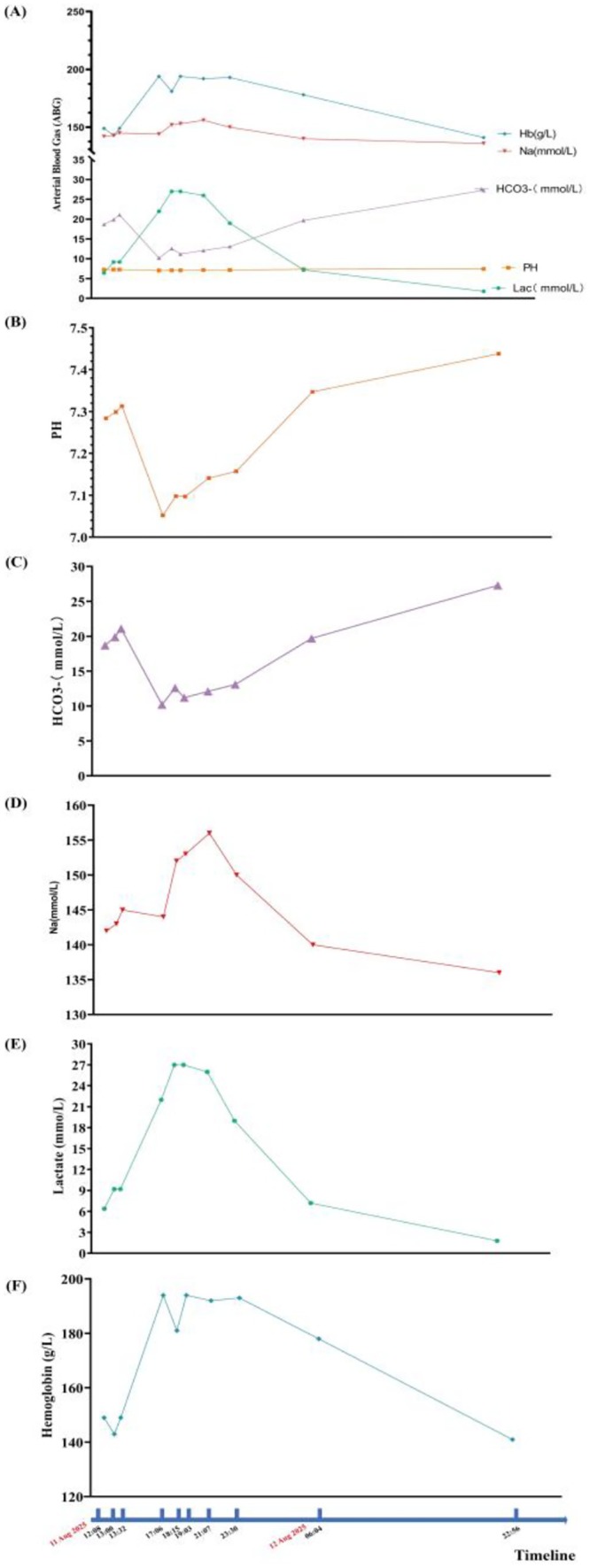
Serial postoperative changes in arterial blood gas and related laboratory parameters. (A) Composite plot summarizing the dynamic changes in hemoglobin, sodium, bicarbonate, pH, and lactate after anesthesia. (B) Blood pH decreased markedly during the shock phase and gradually recovered after supportive treatment. (C) Bicarbonate levels decreased during severe metabolic acidosis and subsequently increased after correction of hypoperfusion. (D) Sodium concentration increased progressively, reaching a peak of 156 mmol/L, and then returned to the normal range. (E) Lactate concentration increased markedly during circulatory shock and decreased after aggressive fluid resuscitation and hemodynamic support. (F) Hemoglobin concentration increased during the hypovolemic phase, consistent with hemoconcentration, and subsequently normalized after fluid replacement.

Postoperative sepsis and septic shock were also considered. The absence of fever was not used as the sole basis for exclusion. However, there was no documented purulent drainage, intraoperative contamination, hypoxemia, abdominal or pelvic tenderness, or imaging evidence of procedure‐related infection or perforation. The clinical course was instead dominated by massive dilute polyuria, rising serum sodium, hemoconcentration, and low filling pressures, which better explained the shock state. Blood cultures were not obtained and empirical antibiotics were not initiated because infection was not considered the leading diagnosis at the time; this represents a limitation of the evaluation. Sodium bicarbonate was administered during profound acidemia as a temporizing measure. We acknowledge that bicarbonate does not treat the underlying cause of hypoperfusion‐related lactic acidosis; definitive treatment in this case was rapid volume replacement and restoration of tissue perfusion. Low CVP and collapsed jugular veins supported a diagnosis of hypovolemia. In the context of severe shock and a lactate level exceeding 20 mmol/L, the patient exhibited marked polyuria (600 mL/h), hypernatremia, and hypotonic urine, with the exclusion of other common causes of DI (e.g., diuretics, intracranial lesions, Diabetic Hyperosmolar State, autoimmune or cerebrovascular diseases). Anesthetic‐associated DI was considered most consistent with the overall presentation. Desmopressin was unavailable at our institution; a brief trial of pituitrin (~2 U) was discontinued because of abdominal distension. Approximately 18 h after onset, urine output decreased to < 100 mL/h, mental status normalized, and serum sodium returned to the normal range (Figures 1, 2, 3; Table 1).

**TABLE 1:** Serial arterial blood gas and laboratory parameters after anesthesia.

Time (after anesthesia)	0.9 h	1.8 h	2.3 h	5.9 h	7.0 h	7.8 h	9.9 h	12.3 h	19.4 h	35.7 h
Lactate (mmol/L)	6.4	9.2	9.2	22	27	27	26	19	7.2	1.8
pH	7.284	7.299	7.313	7.052	7.098	7.097	7.141	7.157	7.347	7.438
HCO_3_^−^ (mmol/L)	18.7	19.9	21.1	10.2	12.6	11.2	12.1	13.1	19.7	27.3
Na (mmol/L)	142	143	145	144	152	153	156	150	140	136
Hb (g/L)	149	143	149	194	181	194	192	193	178	141
Glucose (mmol/L)	12.3	11.1	10.9	11.4	9.5	9.8	11	15.6	14.7	12.4

## 4 Conclusion and Results

Following aggressive fluid resuscitation and hemodynamic support, urine output decreased to < 100 mL/h approximately 18 h after onset. Serum sodium normalized, lactate declined, and mental status recovered. The patient was transferred out of the ICU on postoperative Day 3 and had no recurrent polyuria or electrolyte disturbance during follow‐up.

## 5 Discussion

The present case was characterized by abrupt onset of high‐volume hypotonic polyuria, progressive hypernatremia, hemoconcentration, and subsequent hypovolemic shock shortly after exposure to dexmedetomidine, propofol, and sevoflurane. Although the specific contribution of each agent cannot be determined, the overall presentation was highly consistent with postoperative diabetes insipidus (DI). Proposed diagnostic criteria for postoperative DI include urine output > 300 mL/h for more than 3 h, urine specific gravity < 1.005, and at least one additional feature such as excessive thirst or serum sodium > 145 mmol/L [3]. Our patient fulfilled these criteria, with urine output > 600 mL/h, urine specific gravity of 1.003, urine osmolality of 175 mOsm/kg, marked thirst, and serum sodium rising to 156 mmol/L. In the absence of pituitary disease, intracranial pathology, neurosurgical intervention, or diuretic exposure, a transient anesthesia‐associated disturbance of AVP‐dependent water balance was considered the most likely explanation [1, 2, 3].

The main competing explanations were less consistent with the observed pattern. Osmotic diuresis was unlikely because hyperglycaemia was mild, urine ketones were negative, and the urine was markedly dilute rather than hyperosmolar. Post‐obstructive diuresis was also unlikely, as there was no documented urinary retention or relief of obstruction [4]. In addition, fluid absorption during transurethral endoscopic procedures more typically causes dilutional hyponatraemia and volume expansion rather than hypernatraemic hypotonic polyuria with progressive intravascular depletion [5]. Anaphylaxis was initially suspected because of hemodynamic collapse, but the limited response to epinephrine and corticosteroids, together with sustained dilute polyuria and rising serum sodium, made this explanation less convincing.

Because the patient deteriorated after an endoscopic urologic procedure, surgery‐related complications required careful consideration. Transurethral seminal vesiculoscopy is generally safe, but reported complications include ascending infection/epididymitis, seminal vesicle perforation, and adjacent tissue injury. Accordingly, postoperative hemorrhage, perforation, and early postoperative infection or sepsis were specifically considered in our differential diagnosis.

In our case, hemorrhagic shock was not supported by the laboratory profile, as hemoglobin rose from 145 g/L preoperatively to 194 g/L during deterioration, indicating hemoconcentration rather than occult blood loss. Irrigation‐related complications were also less likely because the patient developed progressive hypernatremia rather than dilutional hyponatremia. Sepsis could not be excluded solely on the basis of afebrile status, since postoperative septic complications may occur without fever; however, the combination of explosive dilute polyuria, low urine specific gravity, rising serum sodium, low filling pressures, and rapid improvement with aggressive volume replacement supported severe free‐water loss as the dominant mechanism of shock in this patient.

Among the agents used, dexmedetomidine has the strongest clinical association with perioperative or sedation‐related DI in the literature [2, 6, 7, 8]. Proposed mechanisms include central suppression of AVP release and reduced AVP‐mediated water reabsorption in the collecting duct [9, 10, 11]. Propofol may also have contributed, as experimental studies have shown inhibition of AVP release from supraoptic neurons, and a recent human case report described transient anesthesia‐associated AVP deficiency supported by serial copeptin and paired serum/urine osmolality measurements [12, 13]. Sevoflurane may have served as an additional contributory factor, as it has been reported to transiently impair urinary concentrating ability, possibly through reduced aquaporin‐2 expression [14]. Given the combined exposure in this case and the similar pattern in prior reports [7], we would avoid assigning causality to a single drug. A more cautious interpretation is transient anesthesia‐associated DI, probably reflecting the combined effects of several agents.

This case has several limitations. Serum AVP or copeptin, serum osmolality, and a formal desmopressin challenge were not available, so central DI could not be distinguished reliably from nephrogenic DI or a mixed disorder. Desmopressin was also unavailable at our institution, and the brief pituitrin trial was not sufficiently informative. Therefore, the causal relationship between the anesthetic regimen and DI should be regarded as suggestive rather than definitive. Despite these limitations, the chronology, the low urine osmolality, the rising serum sodium, and the rapid improvement after supportive treatment make transient anesthesia‐associated DI the most plausible explanation. This case highlights the need to consider DI in patients who develop unexplained postoperative polyuria and hypernatremia, as delayed recognition may rapidly lead to severe hypovolemia and shock.

## Author Contributions

**Yuan Meng:** conceptualization, project administration, writing – original draft. **Xiaoxiao Qu:** data curation, project administration, writing – review and editing. **Chengwei Yin:** methodology, supervision. **Fang Xie:** writing – review and editing. **Yue Jin:** data curation, methodology, supervision. **Geping Zhang:** data curation, supervision. **Rong Shi:** conceptualization, supervision, writing – review and editing.

## Funding

This work was supported by the Shanghai Municipal Key Clinical Specialty (shslczdzk04402) and theScience and Technology Development Project of Shanghai University of Traditional Chinese Medicine (24KFL066).

## Ethics Statement

The authors have nothing to report.

## Consent

Written informed consent was obtained from the patient for publication of this case report and any accompanying images. A copy of the written consent is available for review by the Editor‐in‐Chief of this journal.

## Conflicts of Interest

The authors declare no conflicts of interest.

## Data Availability

Data supporting the findings of this case are available from the corresponding author upon reasonable request.
